# Initiation and propagation of cloud-to-ground lightning observed with a high-speed video camera

**DOI:** 10.1038/srep39521

**Published:** 2016-12-21

**Authors:** M. D. Tran, V. A. Rakov

**Affiliations:** 1 Department of Electrical and Computer Engineering, University of Florida, Gainesville, Florida, 32611, USA; 2 Institute of Applied Physics, Russian Academy of Sciences, Nizhny Novgorod, 603950, Russia

## Abstract

Complete evolution of a lightning discharge, from its initiation at an altitude of about 4 km to its ground attachment, was optically observed for the first time at the Lightning Observatory in Gainesville, Florida. The discharge developed during the late stage of a cloud flash and was initiated in a decayed branch of the latter. The initial channel section was intermittently illuminated for over 100 ms, until a bidirectionally extending channel (leader) was formed. During the bidirectional leader extension, the negative end exhibited optical and radio-frequency electromagnetic features expected for negative cloud-to-ground strokes developing in virgin air, while the positive end most of the time appeared to be inactive or showed intermittent channel luminosity enhancements. The development of positive end involved an abrupt creation of a 1-km long, relatively straight branch with a streamer corona burst at its far end. This 1-km jump appeared to occur in virgin air at a remarkably high effective speed of the order of 10^6^ m/s. The positive end of the bidirectional leader connected to another bidirectional leader to form a larger bidirectional leader, whose negative end attached to the ground and produced a 36-kA return stroke.

The mechanism of lightning initiation remains a mystery, primarily because it usually occurs deep inside the cloud. Most researchers agree that it must involve the creation in the cloud of an elongated ionized region (“lightning seed”) of about 10 m or more in length^1^ that is capable of locally enhancing the electric field at its extremities. Such field enhancement is the main prerequisite for the formation of a hot, self-propagating lightning leader channel. The leader is expected to be bidirectional, although its positive and negative ends may behave differently. Most of the time, bidirectional leaders are completely or in part hidden inside the cloud, which makes their optical imaging impossible. This is why, as of today, there are only a few optical observations of bidirectional leaders in virgin air found in the literature [Montanya *et al*.^2^, Warner *et al*.^3^], and none of them attached to the ground. Further, the positive part of bidirectional leaders is usually “silent” at VHF and, hence, not imaged, because its radiation is significantly weaker than that of the negative part. Also, VHF interferometers are generally unable to resolve sources that radiate simultaneously. As a result, the details of the physics of the bidirectional leader development are presently unclear.

Rison *et al*.^4^ recently suggested that many or possibly all lightning flashes are initiated by the so-called fast (>10^7^ m/s) positive breakdown in virgin air giving rise to narrow bipolar pulses (NBPs), although most of the flashes do not exhibit the NBP-like signature (either wideband or VHF) at their onset. Further, the majority of NBPs observed in Florida are isolated in the sense that their sources, the so-called compact intracloud discharges (CIDs), are not preceded nor followed by full-fledged flashes. Interestingly, the observed fast positive breakdown was apparently not accompanied by the negative breakdown; the latter occurred only after completion of the former. In this scenario, the formation of lightning seed and bidirectional leader is unclear.

In this paper, we present complete evolution of a lightning discharge, from its initiation to ground attachment, which was optically and electromagnetically (RF) observed at the Lightning Observatory in Gainesville (LOG), Florida. This discharge involved a bidirectional leader whose initial extension was predominantly horizontal until the negative end turned toward ground. The dynamics of the positive and negative ends are examined and found to be dramatically different. A simple electrostatic model was used to estimate the electrical characteristics of the bidirectional leader. This study helps to improve our understanding of the initiation of lightning by providing the first high-speed (HS) video records and quantifying the dynamics of a bidirectional leader that resulted in a cloud-to-ground stroke.

## Data Presentation

### Overview

**Figure 1:**
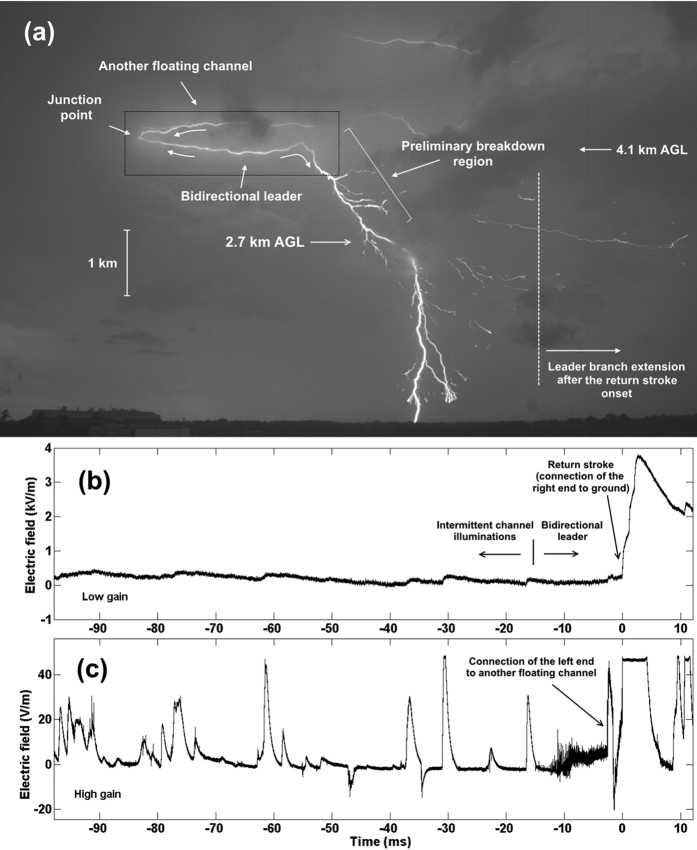
(**a**) Composite image of 41 selected frames (from −123 to 9.8 ms) showing the bidirectional leader, another floating channel, and channel to ground. The high-speed video record started at −178 ms. (**b–c**) Low-gain and high-gain electric field records (from −98 to 12 ms), respectively. No electric fields were recorded prior to −98 ms. The right, negative end turned toward ground, likely due to the presence of positive charge between 4.1 and 2.7 km AGL. The left, positive end of the bidirectional leader made contact with another floating channel (the junction point is labeled in (**a**) and the electric field signature of the connection process is seen in (**c**)) prior to the right end’s making contact with the ground. Note that some leader branches kept extending to the right after the return stroke onset. Individual-frame and composite images of the rectangular area seen in the upper-left corner of (**a**) that show important features of the bidirectional leader development are presented in Fig. 2.

Figure 1a shows a composite image of 41 selected frames of the observed event acquired with the Phantom V310 camera. The frames were selected to accentuate the geometry of the main channel. The left end of the bidirectional leader was identified as positive and the right end as negative (see Supplementary Section S2). All times in this paper are relative to the return stroke onset. According to the HS video images and corresponding electric field records (see Fig. 1b and c), the bidirectional-leader seed was first illuminated 135.2 ms prior to the return-stroke onset, during the late stage of the preceding cloud flash. The initial channel section was intermittently illuminated for over 100 ms, with bidirectional leader extension unambiguously starting at −13.6 ms (maybe even as early as at −14.9 ms). Overall characterization of the thunderstorm based on radar reflectivity and the U.S. National Lightning Detection Network (NLDN) data is given in Supplementary Section S1.

The return stroke occurred about 2.4 ms after the left end had come in contact with another floating channel (see “Junction point” in Fig. 1a). The process of connecting the two floating channels caused saturation of the corresponding frame and, hence, the indicated position of “Junction point” is rather arbitrary. The return stroke was followed by continuing current, whose duration (inferred from high-speed video images) was 21.9 ms.

The NLDN-reported distance from the LOG to the ground strike point is 8.4 km. We used this distance for all points of interest on the luminous channel, which we assumed to be located on the plane that is perpendicular to the camera line of sight and contains the strike point. Based on this assumption, we estimated the distance errors to be between 20% and 30%. All the lengths and speeds presented in this paper are two-dimensional, and, hence, are likely to be underestimates (lower bounds) of actual values. The corresponding three-dimensional values are expected to be about 30% larger^5^,^6^.

We divided the evolution of the observed discharge into 2 stages, based on our optical and electric field records. The first stage, formation of bidirectional leader seed, lasted for about 106 ms (from −135.2 to −28.9 ms). During this stage, there was no detectable channel branching at either positive or negative end; we considered such branching as evidence of leader extension in virgin air. The channel was illuminated intermittently, with the channel brightening being mostly seen in a single frame, followed by channel fading, as expected for recoil leaders retracing pre-conditioned channels. The second stage, extension of bidirectional leader in virgin air, started at −14.9 ms (there was no detectable channel luminosity from −28.9 to −14.9 ms) and ended at the return-stroke onset. Branching at the positive end first occurred at −14.9 ms and at the negative end at −13.6 ms.

**Figure 2:**
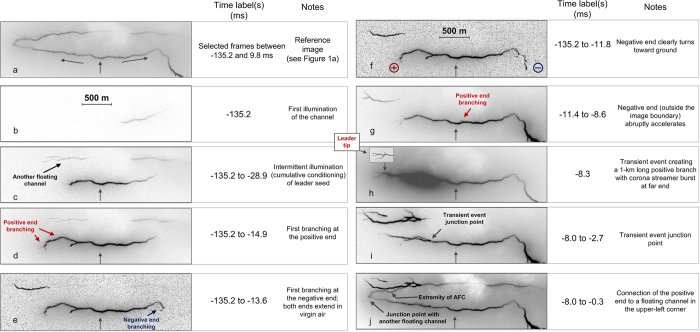
Important features of the bidirectional leader development: (**a**) reference image (same as that shown in the rectangular box in Fig. 1a), (**b**) first illumination of the bidirectional leader channel, (**c**) intermittent channel illumination (another floating channel is seen above the bidirectional leader channel), (**d**) first unambiguous branching in virgin air at the positive end, (**e**) first branching at the negative end (one of the branches is not clearly seen in reproduction), (**f**) negative end clearly turning toward ground, (**g**) abrupt acceleration of the negative end (outside the image boundary), (**h**) transient event creating a 1-km long positive branch with corona streamer burst additionally sketched in the inset to improve visualization, (**i**) transient event junction point, and (**j**) connection of the positive end to a floating channel in the upper-left corner (the frame immediately following the connection is not included in this composite image due to saturation). In (**j**), AFC stands for another floating channel. The vertical upward arrow near a trough-like feature of the channel can be used as a reference in comparing different images. It does not indicate the leader neutral (polarity reversal) point, which is likely to be slightly to the right from the leftward directed branch labeled in (**g**). In (**g**) through (**j**) the negative end is outside the shown image boundary, with both ends being shown in Figs 4 and 5). Time labels correspond to the end of frame exposure times measured with respect to the return stroke onset.

Figure 2a shows, as a reference, the negative of the image seen in the rectangular box in Fig. 1a. Single-frame and composite images for different time intervals are given in Fig. 2b–j to illustrate the salient features of the discharge development, with 2b and 2c corresponding to the first stage.

### Formation of bidirectional leader seed

The “leader seed” (same as “lightning seed”) is defined here as a channel segment that is sufficiently hot and sufficiently long to be polarized and to maintain electrical breakdown at its both extremities. This term was apparently first used by Gurevich and Zybin^7^, who defined it as a “strongly elongated and highly ionized region where conditions for the origin of a streamer are present”. It was also used by Rakov^8^ and Kostinskiy *et al*.^9^. Another term, “plasma patch”, but with a similar meaning was used by Solomon *et al*.^1^.

The first illumination of the prospective bidirectional leader channel (see Fig. 2b) was detected 135.2 ms prior to the return stroke onset at a height of 4.1 km AGL. The illuminated channel segment was 860 m long, although a significant portion of that segment was very faint and hence difficult to see in reproduction. There was no luminosity detected along the first illuminated segment for the preceding 43 ms (since the beginning of the HS video record), so it is likely that it did not carry any significant current, even though it was likely created during the preceding cloud discharge activity. Unfortunately, no electric field signature of the leader-seed creation process is available because the field records began at −98 ms. Using the 2-D length (860 m) of the first visible segment and the interframe interval of 312.5 μs, we estimated the channel luminosity extension speed to be 2.8 × 10^6^ m/s if the extension was unidirectional or 1.4 × 10^6^ m/s if it was bidirectional starting from the mid-point of the imaged segment. These rates are comparable to extension speeds of bidirectional recoil leaders developing along the remnants of positive leader branches. Specifically, mean values of 2-D speeds of negative and positive ends of recoil leaders reported by Warner *et al*.^10^ are 4.53 × 10^6^ and 3.75 × 10^6^ m/s, respectively. Note that the apparent polarity reversal point of the fully developed bidirectional leader was to the left from the image seen in Fig. 2b and that the negative end of a recoil leader is usually observed to be brighter.

Composite image of all frames from −135.2 to −28.9 ms is given in Fig. 2c. During this time interval, the channel was repeatedly illuminated with the lengths of luminous segments ranging from 50 to 1390 m (mean value was 609 m) and the time intervals between illuminations ranging from 8.8 to 29 ms (mean value was 17 ms). These intervals are comparable to the time intervals between K-changes (of the order of 10 ms in a given flash), which are produced by recoil leaders. [Intervals between K-changes in a given channel section are expected to be longer than those between all K-changes in a flash.] It is likely that these intermittent channel illuminations were produced by recoil-leader-type processes, which served to cumulatively condition the leader seed to the point that it could extend in virgin air.

Overall, the discharge activity at this stage appeared as brief sparks migrating throughout the cloud (see Video 1 in Supplementary Section S5) and keeping some segments of the previously created but largely decayed channel network alive. We speculate that when a “weak link” in the channel network is found, it can be converted (via the cumulative effect of multiple sparks following the same path) to a bidirectional leader seed. Once the leader seed is formed, its following bidirectional extension in virgin air is probably independent of its genesis (whether it involved remnants of a previously formed channel, as in our case, or developed entirely in virgin air).

### Development of bidirectional leader in virgin air

At this stage, either the positive or the negative end or both ends of the bidirectional leader exhibited branching, which we assume to be indicative of extension in virgin air. We also assume that the fanning-out type of channel forking (streamer corona burst) can be used for identification of channel extremity. The first unambiguous extension in virgin air was observed at −14.9 ms at the positive end (see Fig. 2d). As noted above, no luminosity along the bidirectional leader channel was seen between −28.9 ms (the end of intermittent channel illumination stage) and −14.9 ms. The negative end started turning toward ground at −14.3 ms. It initially extended as a single channel for 1 ms, which then split into two branches at −13.6 ms. The left branch decayed, while the right one eventually became the main channel of the return stroke. The right branch extended for about 1 ms without pronounced pulses in the high-gain electric field record, possibly due to its predominantly horizontal orientation. The frame-to-frame extension speeds of the negative end between −14.9 and −12.4 ms ranged from 0.33 × 10^5^ m/s to 2.65 × 10^5^ m/s with a mean of 1.67 × 10^5^ m/s.

**Figure 3:**
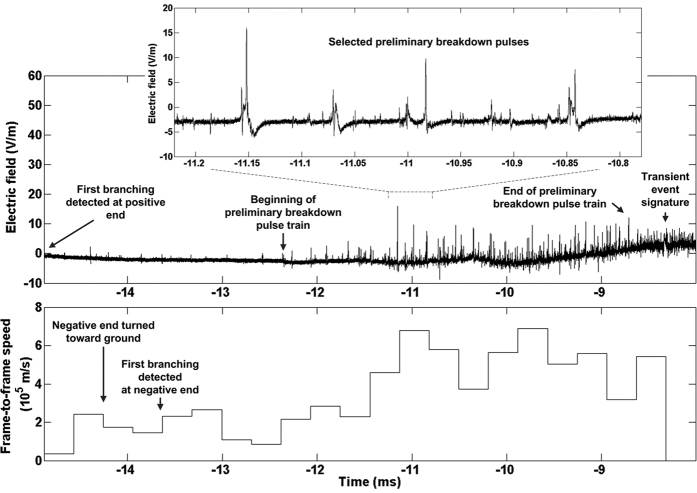
(Top) high-gain electric field record and (bottom) frame-to-frame speeds of the negative end from −14.9 to −8.0 ms. Speed measurements between −8.0 and −6.4 ms were not possible due to obscuration of negative stepped leader by cloud debris.

From −12.4 to −8.6 ms, the negative end of the bidirectional leader exhibited features characteristic of the so-called preliminary breakdown (the process giving rise to a downward stepped leader) in negative cloud-to-ground flashes. Given in Fig. 3 are the high-gain electric field and negative-end frame-to-frame speeds between −14.9 and −8.0 ms, which show the preliminary breakdown pulse train and the corresponding acceleration of the negative end, respectively. For the 68 largest pulses in the train, the mean values of pulse widths and interpulse intervals are 12 and 54 μs, respectively, which are close to their counterparts (16 and 65 μs) reported by Nag and Rakov^11^. The duration of the pulse train is about 3 ms, not far from 3.4 ms, the mean value in Nag and Rakov’s^11^ study. Between −11.8 and −8.6 ms, the negative end significantly brightened and accelerated reaching its maximum 2–D speed of 6.89 × 10^5^ m/s, which is 2.6 times greater than its highest speed (2.65 × 10^5^ m/s) in the −14.9 to −12.4 ms interval. It is likely that the turning of the negative end toward ground and the occurrence of preliminary breakdown process were associated with the presence of a positive charge region between 4.1 km and 2.7 km AGL.

After exhibiting the first branching at −14.9 ms, the positive end was relatively inactive for about 6 ms. Specifically, from −14.6 to −11.4 ms (relatively low negative-end speed), the positive end was largely non-luminous or very faint, and between −11.1 and −8.6 ms (relatively-high negative end speed) it was bright, but appeared not to extend.

**Figure 4:**
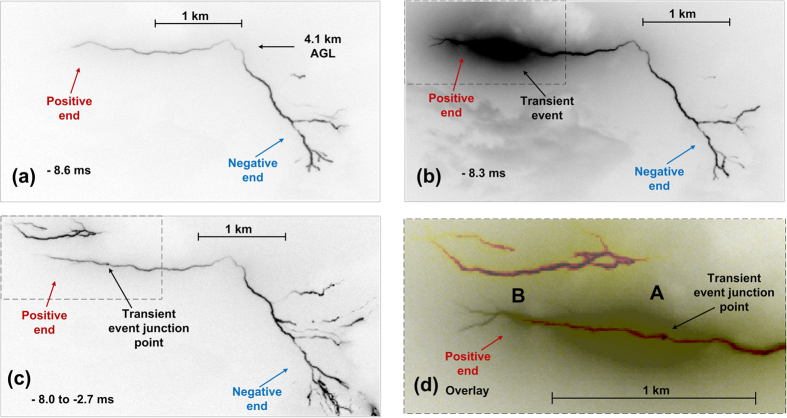
Transient event at the positive end: (**a**) frame at −8.6 ms, just prior to the transient event, (**b**) frame at −8.3 ms containing the transient event, during which a 1-km long, relatively straight branch was formed, (**c**) composite image (from −8.0 to −2.7 ms) showing the transient event junction point, and (**d**) an enlarged view of the overlay of the rectangular areas seen in the upper left corners of (**b**) and (**c**), with the latter being false-color enhanced.

At −8.3 ms, a remarkable event occurred at the positive end, which is described next. A brief and highly luminous process (see Figs 2h and 4b) resulted in the formation of a new, about 1-km long and relatively straight, branch that was forked at its far end. The junction point between this new branch and the previously created channel cannot be seen in Figs 2h and 4b due to light blooming; it was far behind the extremity of the previously formed positive channel, as marked in Figs 2i, 4c and d. The frame-to-frame speed at which the 1-km branch was formed is at least 3.2 × 10^6^ m/s if the luminosity extension was unidirectional and 1.6 × 10^6^ m/s if it was bidirectional, starting from the mid-point of the newly-formed branch. In either case, the speed is remarkably high for a leader developing in virgin air. We term this event transient. The far end of the newly formed branch exhibited four fanning out fingers that are discernible in Video 2 found in Supplementary Section S5 (see also Fig. 2h (in particular the inset), 4b, and 4d), probably resulting from an intense positive streamer corona burst. Interestingly, other positive branches that had been previously visible (see Fig. 2e, f, and g) were not illuminated during this transient event. The transient-event junction point was detectable as a relatively bright spot (see Fig. 4c) in many following frames, until 59 ms after the return stroke, for a total of 67 ms. The transient event was accompanied by a bipolar electric field pulse (marked in the top panel of Fig. 3), whose overall duration was about 60 μs. Its amplitude was not significantly different from that of pulses occurring immediately before and after it, and it was appreciably smaller than the amplitudes of preliminary breakdown pulses, possibly because the resultant branch was predominantly horizontal, while the preliminary breakdown had a significant vertical component. Shown in Fig. 4d is an overlay of the magnified rectangular areas at the upper-left corners in Fig. 4b and c. The purpose of this overlay is to show the position of the junction point within the light-blooming region. False colors are used to enhance contrast between the two overlaid images. The transient event junction point seen in Fig. 4d is clearly off the center of the larger elongated (ellipse-like) light-blooming region, labeled A in Fig. 4d. This may indicate that the junction point seen in Fig. 4c was not the most intense light source in Fig. 4b (during the transient event), possibly suggesting that the new branch evolved from a space leader which collided (more or less in the center of region A) with a relatively short branch originated from the junction point. It appears that there is one more, smaller elongated light-blooming region (labeled B in Fig. 4d), to the left from the larger one. If the light-blooming region is indeed indicative of the collision between oppositely charged channels, then the transient event might have involved two such collisions and, hence, two space stems/leaders. We cannot rule out a possibility that the transient event developed in part along a defunct branch of the preceding intra-cloud discharge, although, if so, it was not sufficiently luminous to be detected for at least 170 ms (starting from the beginning of our optical record).

**Figure 5:**
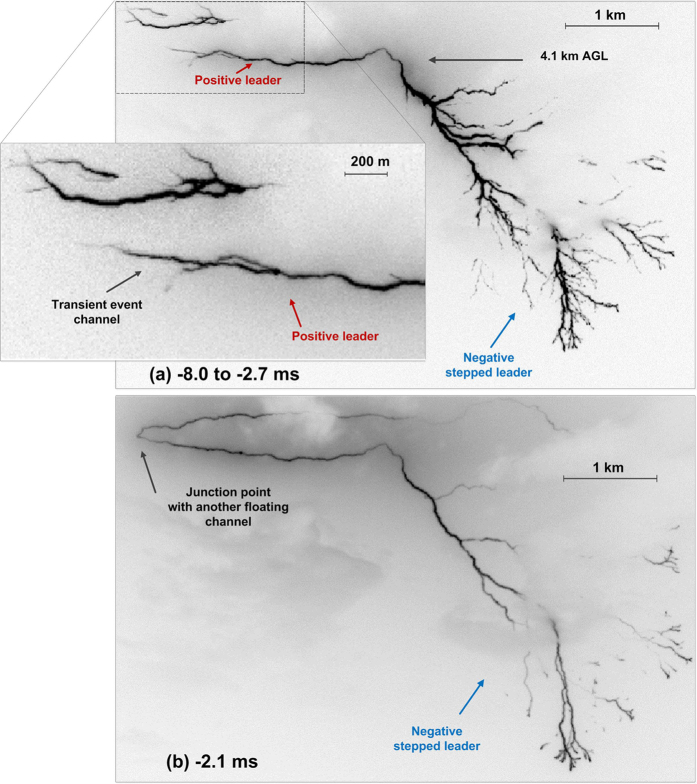
(**a**) Composite image from −8.0 ms (after the transient event) to −2.7 ms (prior to the connection to another floating channel) showing both positive and negative ends of the bidirectional leader. (**b**) Single-frame (−2.1 ms) image showing the enlarged bidirectional leader channel, resulting from the positive end’s (see (**a**)) coming in contact with another floating channel. The precise position of the junction point is unknown and assigned to the abrupt channel kink labeled “Junction point with another floating channel” in (**b**). Frame at −2.4 ms, in which the connection occurred, was saturated. Note that the negative end has a larger extent and is more heavily branched than the positive end.

**Figure 6:**
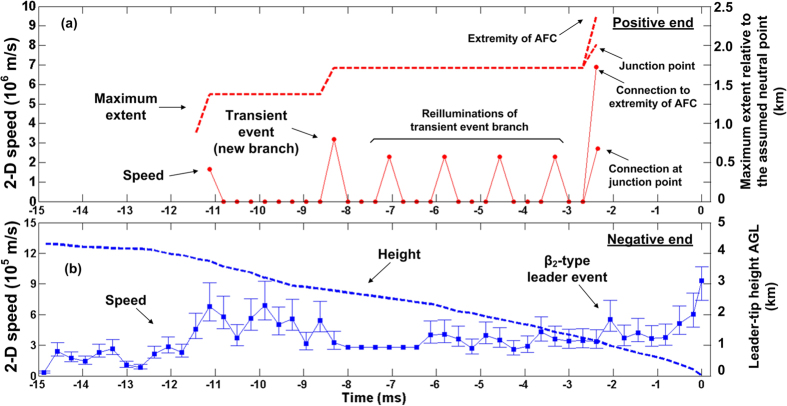
(**a**) Positive leader speed or the speed of intermittent re-illuminations of the transient-event branch (circles and solid line, left vertical axis) and the maximum extent of the positive leader relative to the assumed neutral point (dashed line, right vertical axis) from −11.4 to −2.4 ms. The speed was set to zero when the luminous channel extent was the same as or smaller than in the preceding frame. AFC stands for another floating channel. (**b**) Negative leader speed (squares and solid line, left vertical axis) and leader-tip height AGL (dashed line; right vertical axis) from −14.9 to 0 ms (the return-stroke onset). At about −2.4 ms, connection to another floating channel was established. Speeds of the negative end from −8.0 to −6.4 ms are not available due to channel obscuration by cloud debris and are set to the average speed value determined using the −8.0 ms and −6.4 ms frames. The potential error for the average speed was found to be less than 4%, based on the same approach as that used for speed values evaluated using consecutive frames (see the subsection titled “Development of bidirectional leader in virgin air”). The negative-end speed profile includes the rate of connection of the leader tip to ground, and the positive-end speed profile includes the rate of connection (lower bound) to another floating channel. The maximum extent and speed of the positive end at −2.4 ms in (**a**) are shown for two cases, as if it propagated to the junction point labeled in Fig. 5b and as if it propagated all the way to the extremity of another floating channel (see Fig. 2j). The corresponding maximum extents relative to the neutral point are 2.0 and 2.4 km.

Figure 5a is the composite image from −8.0 to −2.7 ms showing the difference in branching at the positive and negative ends, with the latter exhibiting the typical morphology of negative stepped leader. The difference in the dynamics is quantified in Fig. 6.

Figure 6a shows 2–D speeds of luminous processes at the positive end, which appeared as seven brief luminosity enhancements between −11.1 and −2.4 ms. The 2–D speed profile of the negative end from −14.9 to 0 ms is shown in Fig. 6b. From −14.6 to −11.1 ms, the positive end was largely not visible. Horizontal bars in (b) represent potential errors in speed measurements due to the uncertainties in image occurrence within the frame exposure time. For speeds in (a), only lower bounds could be estimated. The bounds in (b) were computed by assuming that channel tips were imaged by the camera at either the start or the end of frame exposure, and the plot symbols (squares) correspond to the exposure midpoints. The two highest speeds (lower bounds) at the positive end, 3.2 × 10^6^ and 6.9 × 10^6^ (or 2.8 × 10^6^) m/s, were associated with the transient event and the connection to another floating channel, respectively. Other non-zero speeds of the positive end were associated with brief re-illuminations of existing but decayed channel segments, with the transient-event channel being reilluminated four times. We set the speed to zero when the luminous channel extent was the same as or smaller than in the preceding frame. During the four re-illuminations of the transient-event branch, the negative end exhibited speeds ranging from 2.6 × 10^5^ to 6.9 × 10^5^ m/s with a mean of 4.3 × 10^5^ m/s, which is not far from the typical speed of “normal” negative stepped leaders. Our observations suggest that the intermittent luminous processes at the positive end were pumping positive charge into the negative charge source region aloft, while the negative end moved steadily (except for stepping, not resolved in our study) toward ground. Figure 6 also shows the maximum extent at the positive end relative to the assumed “neutral” point and the negative-leader-tip height above ground (see (a) and (b), respectively). The former varies relatively little, while the latter decreases continuously, until the leader tip touches ground and initiates a return stroke. The difference in the behavior of positive and negative ends is dramatic. It appears to be far from sometimes assumed more or less symmetrical double-ended tree growth [e.g., Williams, 2006, Fig. 2; Saraiva *et al*., Figure 20b]^12^,^13^.

It appears from Fig. 6 that the last three brightenings at the positive end were preceded by some acceleration at the negative end (the negative end was obscured prior to the first brightening). There was also acceleration at the negative end prior to the transient event, although two negative-end accelerations between −11 ms and −9 ms were apparently inconsequential. It is also worth noting the negative-end acceleration seen around −2 ms in Fig. 6b. This acceleration could be associated with the connection of the positive end to another floating channel, which resulted in a β_2_-type leader event [Schonland]^14^.

At −8.3 ms, the tip of the transient-event branch was marked by the fanning-out forking (see the inset in Fig. 2h), which defined the maximum detectable extent of the positive end before its making contact with another floating channel at −2.4 ms. Between −8.3 and −2.4 ms, the transient-event branch appeared to be the only active one at the positive end. It repeatedly faded and brightened, exhibiting a kind of pulsating behavior. No steady channel extension was detected. If such extension were present, its average speed between −8.3 and −2.4 ms would be 4.6 × 10^4^ m/s if the transient-event branch extended to the junction point and 1.1 × 10^5^ m/s if it extended all the way to the extremity of another floating channel (see Fig. 2j). The lower speed value, corresponding to the more likely scenario, is of the same order of magnitude as those expected for steadily-extending, in-cloud positive leaders (e.g., Williams^12^ [section 5.2.2] and Saraiva *et al*.^13^ [section 3.1.2]). If indeed there was a relatively slow, steady elongation of the positive end between −8.3 and −2.4 ms, the speeds shown in Fig. 6a should be viewed as corresponding only to the intermittent re-illuminations of the transient-event branch. Note that the speeds shown in Fig. 6a before −8.6 ms correspond to a different positive-leader branch.

At about −2.4 ms, the positive end of the bidirectional leader connected, via the branch created during the transient event, to another floating channel to form a larger bidirectional leader, as seen in Fig. 5b. The junction point could be anywhere between the tip of the transient event channel and the extremity of the other floating channel. We assume that the junction point was at the channel kink located 270 m to the left from the tip of the transient-event channel and marked in Figs 1a, 2j and 5b.

## Discussion

We presented the first optical observation of a bidirectional leader giving rise to a negative stepped leader/return-stroke sequence. The negative end exhibited optical and RF electromagnetic features characteristic of negative preliminary breakdown and stepped leader, while the positive end exhibited a kind of pulsating behavior. These pulsations can be seen in the video record (Video 2) found in Supplementary Section S5. See also the “triangular pulses” in the speed profile shown in Fig. 6a.

The bidirectional leader extension normally occurs inside the cloud (typical cloud-base height in Florida summer storms is 1–1.5 km), with only the downward part developing below the cloud base. The observed event occurred near the periphery of a compact, high-radar-reflectivity cell, so that most of its luminous channels were not significantly obscured by the cloud or cloud debris.

The positive end of the bidirectional leader was predominantly horizontal and located at relatively low altitudes of about 4 km. It could be viewed as supplying negative charge to the negative end from those altitudes. This is consistent with the fact that the thunderstorm was in its dissipating stage and with MacGorman *et al*.’s^15^ finding that a negative charge layer in dissipating Florida thunderstorms is found near and just above the 0 °C level, which in our study was between 4 and 5 km AGL.

We inferred the occurrence of preliminary breakdown process at altitudes ranging from 4.1 to 2.7 km (after the negative end turned toward ground), which, as hypothesized by Nag and Rakov^16^, indicates the presence of a significant positive charge layer immediately below the negative one. Such a positive charge layer was observed by MacGorman *et al*. [Fig. 2]^15^ in one of the three balloon soundings of Florida dissipating storms.

It isn’t clear if the polarity reversal (neutral) point was stationary during the bidirectional leader development. Based on the direction of channel branching, we infer that the neutral point was slightly to the right from the leftward-directed (positive) branch labeled in Fig. 2g. That point was repeatedly traversed by fast luminosity waves, which is in contrast with the event reported by Montanya *et al*.^2^, who inferred from their high-speed video record that the neutral point was stationary. It is important to note that the two leader ends in Montanya *et al*.’s^2^ case started their extension at the same time from a single point in virgin air. In contrast, in our case, a decayed channel section was involved. Additional comparison with Montanya *et al*.’s^2^ event is found in Supplementary Section S3.

Our views of bidirectional leaders in general are as follows. Leaving aside bidirectional leaders originating from metallic objects^17^,^18^,^19^, there appear to be four main scenarios for the formation of bidirectional leaders: (1) initiation of lightning in the cloud [present study; see also Kostinskiy *et al*.^9^,^20^], (2) lightning channel branching process [Montanya *et al*.^2^; Warner *et al*.^3^], (3) space leader involved in the negative leader step-formation process [Gorin *et al*.^21^; Gamerota *et al*.^22^], and (4) recoil-leader-type process giving rise to K-changes, dart leaders, and M-components [e.g., Warner *et al*.^10^; Mazur *et al*.^23^]. The formation of bidirectional leader may occur in virgin air (scenarios 1, 2, and 3) or in decayed channel branches (scenario 4). In the latter case, electrical breakdown is easier to initiate, since it occurs in warm, low-density air which the decayed lightning channel branches are filled with. Creation of bidirectional leader seed in scenario 1 may involve a decayed channel section, with the following extension taking place in virgin air [present study]. The dynamics of positive and negative leader ends in terms of the extension speed and branching can be very different either for the same bidirectional leader or for leaders following different scenarios. Positive end can extend faster^22^,^24^ or slower^2^,^3^ than the negative end, or the two ends can extend at similar speeds^10^. In the present study, we observed a pulsating behavior of the positive end, while the negative end extended normally. The spatial scale can vary from meters^22^ to many kilometers [present study]. Recoil-leader-type processes usually occur in decayed positive leader branches, but can also be formed in decayed negative branches, as recently reported by Montanya *et al*.^25^ and Stolzenburg *et al*.^26^.

Our transient event exhibited some similarities with the so-called restrike phenomenon, that was previously reported from long positive laboratory spark experiments. The term “restrike” was introduced by Les Renardieres Group^27^,^28^ who studied positive laboratory sparks in 5 or 10 m gaps with different high-voltage electrode shapes (hemisphere, hyperboloid, or cone). They reported abrupt increases in the positive leader channel length that was accompanied by a vigorous corona streamer burst at the leader tip. At the same time the voltage drop along the leader channel decreased due to dramatic increase in channel conductivity.

It has been found by Les Renardieres Group that the probability of restrikes increases with absolute humidity; the restrikes cause a sharp increase in overall mean leader speed when the humidity exceeds 10 g/m^3^. The leader channel extension can stop after a large restrike due to the injection of significant positive space charge into the gap by the corona streamer burst. Recent observations of positive leader restrikes under high humidity conditions (15.5–16.5 g/m^3^) were reported by Chen *et al*.^29^. They found the restrike speeds to be 0.5 to 2.0 × 10^5^ m/s vs. 2.2 × 10^4^ m/s during the “stable stage” of leader development. Some restrikes in their study exhibited structured (branched type, as opposed to diffuse) streamer zones. From two National Weather Service sounding balloon measurements 112 km from the LOG, the pressure at the ground level was 1014 hPa, and the absolute humidity was found to be 20 to 24 g/m^3^ at 12:00 to 24:00 on the same day (UTC), respectively. At 4 km AGL, the absolute humidity was about 5 g/m^3^.

During our transient event, the rate at which the new positive branch was formed was at least 3.2 × 10^6^ m/s if the extension was unidirectional and 1.6 × 10^6^ m/s if it was bidirectional starting from the mid-point of the newly-formed branch. In either case, the speed is remarkably high for a leader developing in virgin air (there was no luminosity detected along its path for at least 170 ms, since the beginning of the optical record). There are only a few observations/inferences of such high leader speeds in virgin air found in the literature. Yoshida *et al*.^30^ reported upward positive leader (UPL) speeds of the order of 10^6^ m/s (the corresponding leader current was of the order of kiloamperes) in rocket-triggered lightning, and Zhu *et al*.^31^ inferred similarly high speeds for exceptionally fast negative stepped leaders initiating return strokes with peak currents in excess of 100 kA in natural lightning. Interestingly, the channel-base current of Yoshida *et al*.’s UPLs^30^ exhibited pulsations which are suggestive of stepping process. Perhaps our transient event can be considered as a gigantic, kilometer-scale positive-leader step that developed from a space stem/leader and connected to the existing channel, creating a major branch which eventually became part of the main channel. The transient event could have injected a significant amount of positive charge near the newly-formed positive end (the fanning-out branches at the extremity of the transient-event channel (see inset in Fig. 2h) are probably the most intense streamers of the corona streamer burst). This space charge could have inhibited further extension of the positive end, as was observed in long positive sparks, with the likelihood of overcoming the space charge effect being higher for higher rate-of-rise of voltage across the gap.

Besides the transient event which attempted to forge a path to another floating channel, we observed four abrupt brightenings of the transient-event branch (see the four accelerations following the transient event and separated by 1.2 ms in Fig. 6a). Only after those four additional attempts connection to another floating channel was established. The development of a bidirectional leader can be viewed as an elongating-conductor polarization process in an external electric field. In this regard, the dynamics of the positive end should be coupled to those of the negative end. In our case, it appears that the negative leader stepping (on a microsecond time scale) and slow (millisecond scale) negative charging process result in a relatively slow potential rise at the positive end, which is insufficient for continuous extension of that end and causes the intermittent (on a millisecond time scale) pumping of positive charge into, or draining negative charge from, the source region.

Below we briefly discuss a simple electrostatic model of our bidirectional leader (more details are found in Supplementary Section S4), applied to the time interval between −11.1 and −2.7 ms; that is, to the time interval during which the positive end could be reasonably well tracked (see Fig. 6a). We represented the channel (including branches) by three straight sections, top horizontal, middle tilted, and bottom vertical (see Supplementary Figure S2). The following assumptions were made. First, the neutral point (shown in Supplementary Figure S2) was stationary. Second, the line charge density along either positive or negative channel increased linearly from the neutral point (where it is zero) toward the far end. Third, the negative end extended at a variable speed estimated from the high-speed video images (see Fig. 6b) and at a constant charge density slope, while the positive end had a constant spatial extent (as seen in Fig. 6a, neglecting the possible existence of undetected very faint channels) and a charge density slope increasing with time (to satisfy the principle of conservation of charge). The negative charge density slope was selected to match the measured net leader electric field change from −11.1 to −2.7 ms. The net charge on the bidirectional leader channel was zero at all times.

At −2.7 ms, the maximum magnitudes of charge density (at the extremity) of negative and positive leaders were found to be 1.6 and 5.5 mC/m, respectively. The charge transfer between −11.1 and −2.7 ms was 3.3 C, and the corresponding average current was 393 A. It was also found that uncertainties in the height of the horizontal channel section and in the location of the neutral point did not significantly affect the computed charge transfer value. If the slope of negative charge density is assumed to remain the same until the leader attachment to ground and the increase of positive end length due to connection to another floating channel is neglected, the total charge transfer *Q* will be 5.6 C. This is the charge deposited on the negative part of the bidirectional leader. If we assume that the charge neutralized by the return stroke is equal to that deposited on the negative part of bidirectional-leader channel, then according to the empirical formula relating the impulse charge transfer to the return-stroke peak current, *I* = 10.6*Q*^0.7^ [Berger]^32^, the corresponding peak current will be 35 kA. The latter value is close to 36 kA estimated from radiation magnetic field peaks measured at multiple NLDN stations for the return stroke initiated by the modeled bidirectional leader.

## Summary

- We presented the first optical observation of lightning discharge in its entirety, including its initiation, propagation, and attachment to the ground. The leader seed of some hundreds of meters in length was formed, after cumulative conditioning for over 100 ms, in a decayed channel section of the preceding cloud flash. Then the leader seed evolved into a bidirectional leader that extended in virgin air for at least 11 ms.

- The negative end of the bidirectional leader exhibited characteristic features of preliminary breakdown and stepped leader in negative cloud-to-ground strokes.

- The behavior of the positive end was dramatically different from that of the negative end. The positive end appeared to be inactive or pulsating vs. relatively steady extension of the negative end. Specifically, the positive end exhibited an abrupt and relatively straight extension of about 1 km in virgin air with a streamer corona burst at its far end. This newly-created branch faded and then was re-illuminated fours times with a remarkably constant time interval of 1.2 ms. All the five jump-like events at the positive end probably served to pump positive charge into (or drain negative charge from) the cloud source region.

- Using a simple electrostatic model, we estimated the magnitude of charge deposited on either positive or negative part of the bidirectional leader channel to be 5.6 C, which statistically corresponds to the return-stroke peak current of about 35 kA. The latter value is close to 36 kA which was estimated by the NLDN from return-stroke magnetic field peaks measured at multiple stations, separated on average by 300–350 km.

## Methods

The LOG is located on the roof of a five-story building on the campus of the University of Florida. The instrumentation setup presently includes high-speed (HS) video cameras, low-gain and high-gain wideband electric field, electric field derivative (d*E*/d*t*), and magnetic field derivative measuring systems, and an x-ray detector. The flash studied here was captured by two high-speed cameras (Phantom V310 and Megaspeed HHC-X2; only the Phantom record is shown in this paper). The corresponding electric field and d*E*/d*t* records were also obtained. The magnetic field derivative signals were not recorded and no x-rays were detected from this flash. The useful bandwidth of the low-gain electric field measuring system was 16 Hz to 10 MHz. The instrumental decay time constant was 10 ms. For the high-gain system, the bandwidth was from 360 Hz to 10 MHz, and the decay time constant was 440 μs. The upper frequency response of the d*E*/d*t* measuring system was 10 MHz. The field measuring systems were synchronized with Phantom V310 and HHC-X2 cameras with accuracy better than 1.3 μs and 1 ms, respectively^33^. The Phantom V310 camera was coupled with a Sigma 20 mm lens, whose f-number (f-stop) was set to f/4. The camera framing rate was 3200 frames per second with the exposure time of 80 μs and dead time of 232.5 μs. The spectral sensitivity was between 40% and 30% quantum efficiency (QE) for wavelengths from 425 to 750 nm, and 20–30% QE from 750 to 810 nm. A C-mount fisheye lens was used for the HHC-X2 camera. The f-number was set to f/4 and the framing rate was 1000 frames per second with the exposure time of about 1 ms. The deadtime of the HHC-X2 camera was negligible compared to its exposure time. The pre-trigger time of the field measuring system was 100 ms, and for both cameras it was 200 ms. The electrostatic model used to estimate electrical parameters of the event observed with the instrumentation described above is presented in Supplementary Section S4. We additionally used data from the U.S. National Lightning Detection Network (NLDN), as well as the National Weather Service radar (see Supplementary Section S1) and balloon observations near Jacksonville, FL, 112 km from the LOG.

## Additional Information

**How to cite this article**: Tran, M. D. and Rakov, V. A. Initiation and propagation of cloud-to-ground lightning observed with a high-speed video camera. *Sci. Rep.* **6**, 39521; doi: 10.1038/srep39521 (2016).

**Publisher's note:** Springer Nature remains neutral with regard to jurisdictional claims in published maps and institutional affiliations.

## Supplementary Material

Supplementary Information

Supplementary Video 1

Supplementary Video 2
