# Supplementary material for: Initiation and propagation of cloud-to-ground lightning observed with a high-speed video camera
Source: Sci Rep. 2016 Dec 21;6:39521. doi: 10.1038/srep39521 (PMC5175180; doi:10.1038/srep39521)
Supplement: Supplementary Information [file srep39521-s1.pdf]

Supplementary Information to "Initiation and propagation of cloud-to-ground lightning observed with a high-speed video camera"

M.D. Tran[a], V.A. Rakov[a,b]

[a]*Department of Electrical and Computer Engineering, University of Florida, Gainesville, Florida, 32611, USA*
[b]*Institute of Applied Physics, Russian Academy of Sciences, Nizhny Novgorod, 603950, Russia*

S1. Characterization of the thunderstorm that produced the bidirectional leader making contact with ground

At about 20:03 UT (16:03 local time) on 2 August 2014, a cluster of about 8 thunderstorm cells in a large (about 100 km in extent) thunderstorm system crossed the Gainesville area, moving from southwest to northeast. The horizontal extent of the cell cluster (with radar reflectivity $\geq$ 35 dBZ and at an altitude of 5 km above ground level (AGL)) reached its maximum of about 44 km at 21:54 (UT). Its 35-dBZ upper boundary was higher than 10 km AGL. At about 21:49 (UT) the flash rate estimated from NLDN records (within 30 km of the NLDN-reported ground termination of the flash examined in this study) reached its peak of 19 flashes per minute. Afterwards, the cell cluster was diminishing in horizontal extent and its upper boundary was descending. At 22:45 (UT) the 35-dBZ cloud top was at about 7 km AGL and the cluster disintegrated into 4 isolated cells, each being smaller than 8 km in horizontal extent (with $\geq$ 35 dBZ reflectivity and at a height of 5 km AGL). The 0°C temperature level was 4.8 km AGL, according to two balloon sounding measurements at 12:00 (UT) and 24:00 (UT) on 2 August 2014 at the weather station in Jacksonville.

At 22:43:33 (UT), when the thunderstorm was in its dissipating stage (the flash rate dropped to 4 flashes per minute), it produced a clearly documented bidirectional leader that evolved into a single-stroke, negative cloud-

*Corresponding authors
Email addresses: manhtran@ufl.edu (M.D. Tran), rakov@ece.ufl.edu (V.A. Rakov)

to-ground flash. The data acquired at the LOG are summarized in Figure S1 together with the NLDN data.

The NLDN reported a total of 7 events from -522 to 1.2 ms (relative to the return-stroke onset), including a 36-kA cloud-to-ground (CG) stroke and 6 cloud pulses, 5 preceding and 1 following the CG stroke (the latter pulse is not further discussed in this paper). Of the 5 cloud pulses preceding the CG stroke, 4 occurred within less than 2.7 km of each other, in a compact region with radar reflectivity exceeding 26 dBZ at heights ranging from 1 to 4 km AGL. A portion of the high radar reflectivity region apparently corresponded to the denser cloud region seen in Figure 1a of the paper to the right from the bidirectional leader channel. The event presented here was just outside that high-reflectivity region with only some cloud debris relatively insignificantly obscuring its development. This is confirmed by more or less uniform luminosity of the main channel during the continuing current stage following the return stroke. The positions of cloud debris were well known from the analysis of the video record at different stages of the discharge characterized by different levels of light intensity (see Figure 5 of the paper). The sources of the 4 cloud pulses were at horizontal distances of 8.2-11.1 km from the channel to ground, the duration of the four-pulse sequence was 64 ms, and the time interval between the last reported pulse in the sequence and the return stroke was 458 ms. The fifth NLDN-reported cloud pulse (not shown in Figure S1) occurred 319 ms prior to the return stroke and 5.4 km from its ground termination.

S2. Identification of polarity of bidirectional leader ends

After the connection of the left end of the bidirectional leader to another floating channel at -2.4 ms, the right end contacted ground (at $t = 0$) and produced a negative return stroke. Therefore, the right end after the connection must have been negative. We argue below that the right end was also negative prior to the connection of the left end to the floating channel.

1. From -12.4 to -8.6 ms, the right end accelerated, vigorously branched, and brightened in virgin air, while the left-end luminosity was retracing the previously-created channel and showed very little overall extension (see Figures 2f and 2g of the paper), until the transient event at -8.3 ms. Therefore, the observed pronounced preliminary breakdown pulses (see Figure 3 (top panel) of the paper) are to be attributed to the right end. Since, the preliminary breakdown pulses are positive (same polarity as that of the following return-stroke pulse), we conclude that the right

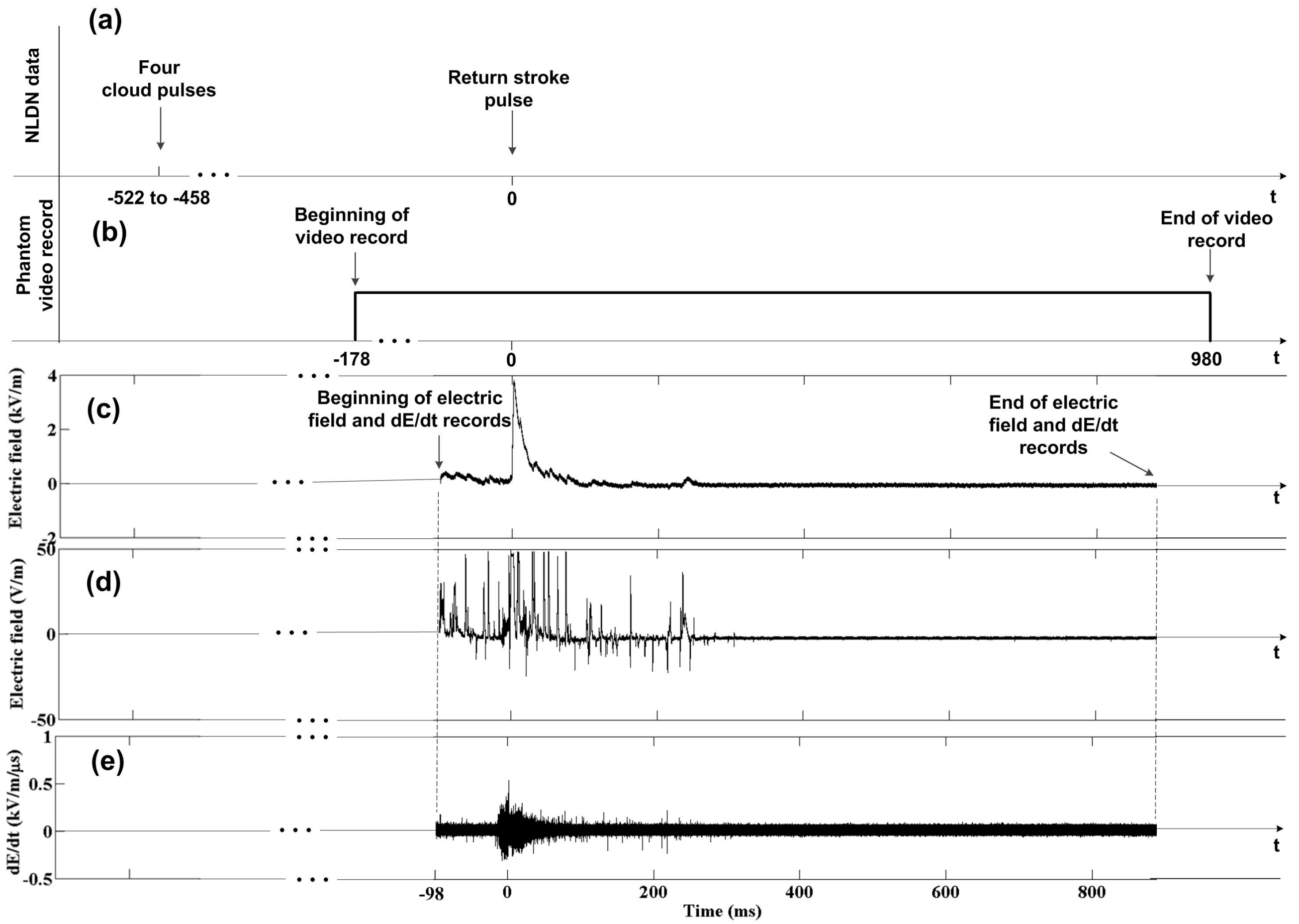

Figure S1: Summary of records used in this study. (a) Cloud pulses and the return-stroke pulse reported by the NLDN. (b) High-speed video images that are available from -178 to 980 ms. (c and d) Low-gain and high-gain electric field records, respectively, and (e) dE/dt record. The field records are available from -98 to 902 ms. $t = 0$ is the return-stroke onset time.

end was negatively charged prior to the connection of the left end with the floating channel.

2. Figure 5a of the paper is the composite image (from -8.0 to -2.7 ms) of bidirectional leader prior to the connection of its left end to the floating channel. The morphology of the right end in Figure 5a of the paper is characteristic of negative stepped leaders, which is in support of the above inference that the right end was negative prior to the connection of the left end with the floating channel.

S3. Polarity asymmetry

Williams [S1] reviewed various aspects of polarity asymmetry in lightning observations. Regarding bidirectional leaders, he stated that the positive and negative ends "propagate at different speeds, with different degrees of steadiness, and with different radiated electromagnetic energy". We do concur with this statement. It is generally not easy, however, to quantify those differences due to the lack of adequate experimental data.

In the following, we compare our observation of bidirectional leader with the one reported by Montanya et al. [S2]. Both observations show a clear difference in branching at the two ends, with the negative end being more heavily branched. There are, however, a number of differences between the two bidirectional leader observations.

The most important differences are related to the initiation and "final destination" of the bidirectional leader. Montanya et al.'s [S2] event started abruptly from a single point in space and connected, via its negative end, to the existing cloud discharge channel. Our event was preceded by about 106 ms of intermittent illuminations that apparently conditioned the leader seed whose length was some hundreds of meters. Further, the event studied here first made connection, via its positive end, to another floating channel and then, via its negative end, to ground and produced a 36-kA negative return stroke.

The positive end in our case exhibited the first extension and branching in virgin air well (1.3 ms) before its negative counterpart did. The two leader ends in Montanya et al.'s case [S2] started their extension at the same time. The negative end was heavily branched, while the positive end remained a single channel for about 6 ms until the bidirectional leader connection to another "pre-existing positively charged lightning channel".

The positive end of the event presented here exhibited a kind of pulsating behavior. From the location of the rightmost positive branch (labeled in

Figure 2g in the paper), the maximum extent of the positive part was found to be about 2 km, which is considerably shorter than the 6.6-km maximum extent estimated for the negative part. The most remarkable extension of the positive end was during the transient event resembling a gigantic step. It was not associated with the existing positive leader tip and apparently occurred in virgin air at a relatively high speed of 3.2×10^6 m/s (if the extension was unidirectional) over a relatively large distance of 1 km. The relatively small extension of the positive end (apart from the transient event) is puzzling and in contrast with previous studies. For example, the two ends of Montanya et al.'s [S2] event extended continuously with the speed of the positive end being one-half of that of the negative end. Also, , van der Velde and Montanya [S3], from a Lightning Mapping Array (LMA) study of natural lightning flashes in Spain, concluded that negative and positive leaders propagated at characteristic horizontal speeds of 10^5 and 2×10^4m/s, respectively, and that "positive leaders remain propagating throughout the flash or until reaching ground". The "horizontal speeds" of lightning processes were found using the so-called time-distance graph in which the variation of horizontal (radial) distance between LMA sources and a reference point (often chosen as the flash origin) is plotted vs. time. The horizontal speed is defined as the slope the time-distance graph. Note that for estimating the speed of positive leaders, which are usually not imaged by LMA, the sources of negative breakdown retracing decayed positive breakdown channels were used.

S4. Electrostatic model of bidirectional leader

The electrostatic model described here represents the development of our bidirectional leader between -11.1 and -2.7 ms (note that connection of the positive end to another floating channel occurred later, at about -2.4 ms). Figures S2a and S2b are the composite images of frames before and during the time interval of interest. The corresponding channel representations in the model are shown in Figures S2c and S2d. The maximum extent of the positive end appeared to vary only slightly, while the negative end extended steadily (see Figure 6b of the paper) in the 4.1 to 1.0 km height range.

Our electrostatic model is based on the assumptions given below.

1. The intermittent, predominantly horizontal cloud discharges that occurred during the time interval of interest insignificantly contributed to the electric field on the ground surface. This assumption is based on our modeling results which show that short horizontal floating channels produce negligible vertical electric field change at the ground level.

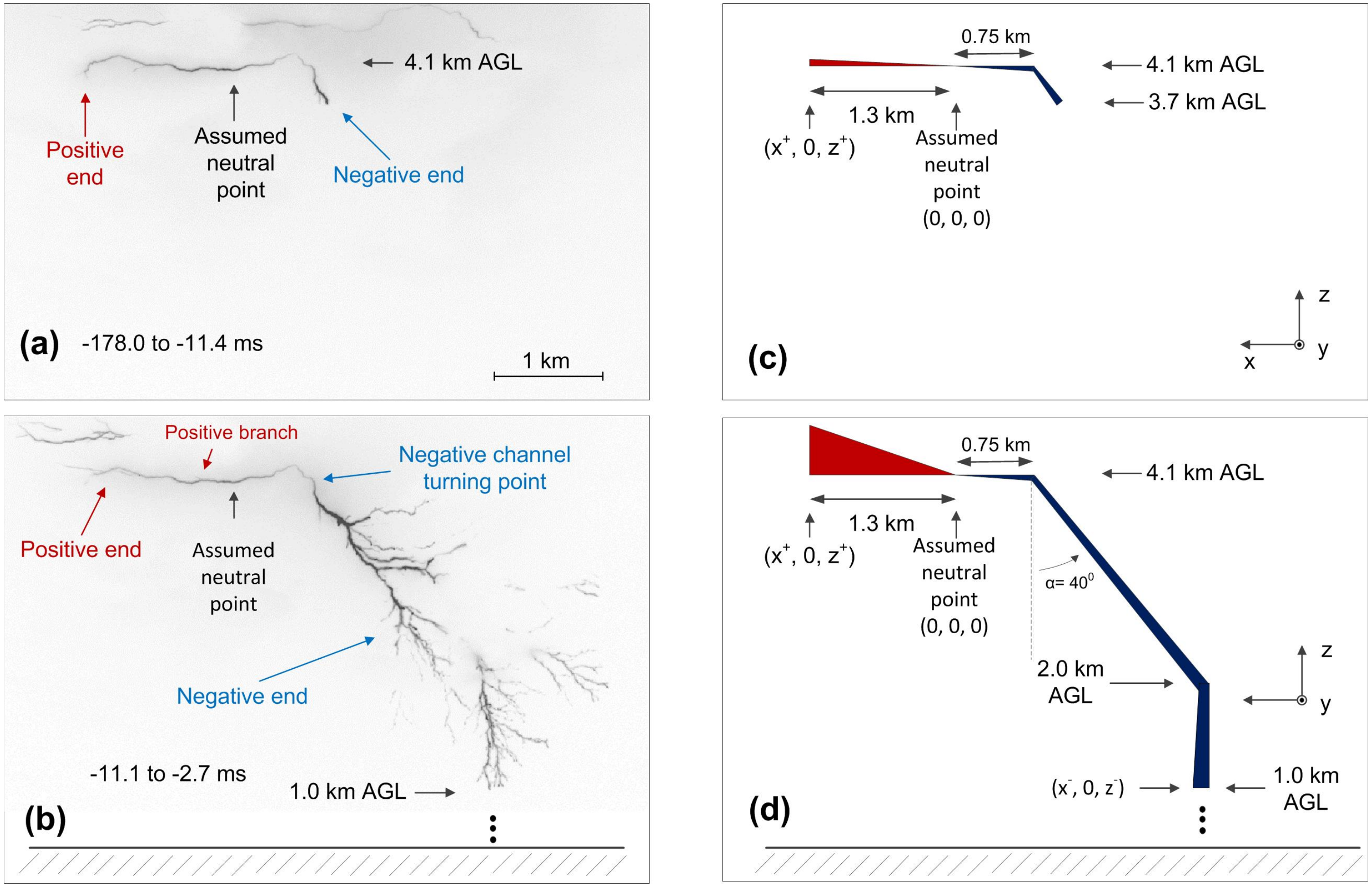

Figure S2: (a) Composite image of all frames between -178 ms (the beginning of the high-speed video record) and -11.1 ms. (b) Composite image of frames between -11.1 and -2.7 ms (frame -8.3 ms containing the transient event (see Figure 2h of the paper) is excluded), showing the negative end descending from 4.1 to 1.0 km. (c and d) Geometries of our electrostatic model at -11.1 and -2.7 ms, respectively. The neutral point is assumed to be stationary between the rightmost positive branch labeled in (b) and the ground-bound turn at the negative end. The line charge density is assumed to be linearly increasing from zero at the assumed neutral point to maxima at the extremities of the positive and negative leader channels.

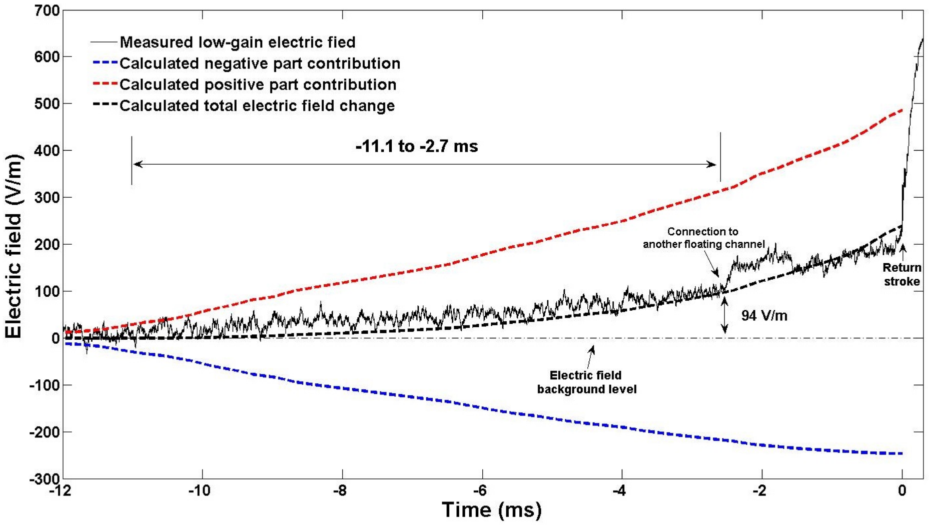

Figure S3: Measured electric field (after compensation for instrumental decay) at 8.4 km vs. electric fields computed using the electrostatic model. The net electric field change between -11.1 and -2.7 ms is 94 V/m.

For example, a 2-km long, horizontal bipolar channel, whose charge density slope is similar to that of the bidirectional leader (will be discussed later) produced a vertical electric field change of about 0.7 V/m vs. the measured net field change of 94 V/m (see Figure S3).

2. The polarity reversal (neutral) point was stationary between -11.1 and -2.7 ms and was located somewhere between the short positive branch labeled in Figure S2b and the first ground-bound turn of the negative end. In the model, we assumed that the neutral point was located at 0.75 km to the left from the negative-end turning point (see Figures S2c and S2d). The variation of the neutral-point location within the limits indicated above resulted in less than 9% difference in the total charge transfer.

3. The positive part of the bidirectional leader was essentially horizontal between -11.1 and -2.7 ms (see Figure S2b) and its length was set to 1.3 km. In order to satisfy the principle of conservation of charge, the slope of positive line charge density was made variable.

4. The negative part consisted of a horizontal, tilted, and vertical sections. The tilted section extended at angle $\alpha = 40°$ with respect to

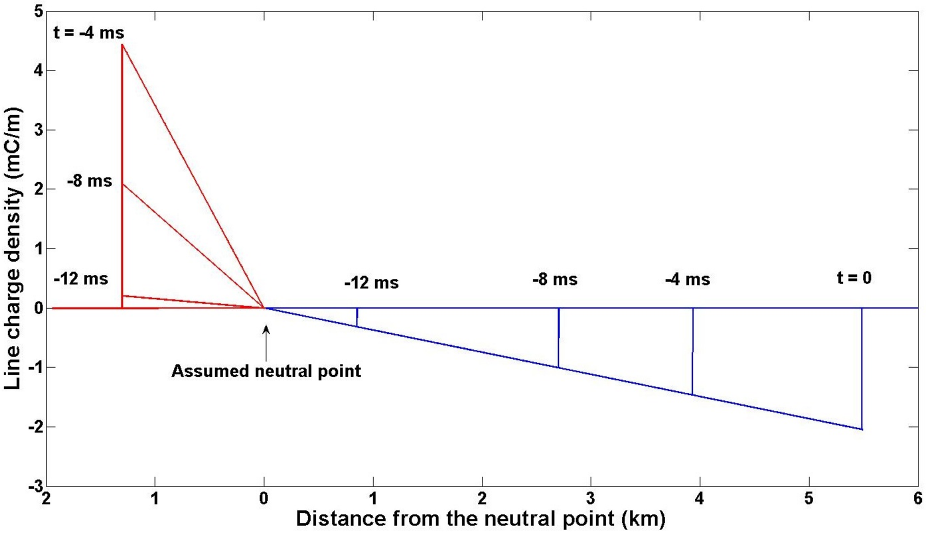

Figure S4: Line charge density vs. scalar distance from the neutral point. Red and blue lines correspond to the positive and negative parts of the bidirectional leader, respectively, at 4 (negative end) or 3 (positive end) instants of time, starting from -12 ms with a time step of 4 ms. The slope of line charge density at the negative end is constant, while at the positive end it increases to keep the net charge on the leader channel equal to zero at all times. The positive line charge density at $t = 0$ is not shown because at -2.4 ms the positive end connected to another floating channel of unknown length.

vertical. Its junction point with the lowest, vertical section was 2 km AGL (see Figure S2d). The channel extension speeds are the frame-to-frame speeds estimated from the HS video record (see Figure 6b of the paper). The slope of line charge density along all three negative channel sections was assumed to be the same and not change with time from -11.1 to -2.7 ms.

5. The line charge density of the positive and negative parts was zero at the neutral point and linearly increased toward each of the channel extremities. The net charge on the entire bidirectional leader channel was zero at all times.

6. The transient-event channel increased the 2D length of the positive part by 0.35 km, which was neglected since it resulted in less than 5% difference in the total charge transfer.

Figures S2c and S2d show the (x, y, z) coordinates of the extremities of the positive and negative parts, which are $(x^+, 0, z^+)$, and $(x^-, 0, z^-)$, respectively, all being in the xz vertical plane at $y = 0$. The origin of coordinates is set to the neutral point. z_0 and x_0 are the height of the neutral point and its distance to the ground-bound turning point of the negative end, respectively, which were varied to study the effect of uncertainties in distance measurements and in the assumed neutral point location.

In our model geometry shown in Figure S2, $z_0 = 4.1$ and $x_0 = 0.75$. The observation point is located at (-0.75, 8.4, -4.1) at the ground level, 8.4 km from the vertical plane. The coordinates of different points of interest including the extremities of the positive and negative ends at different times are given in Table S1. The time step is set to 1 μs, so the length of leader segments, which were approximated by point charges, varied from 0.03 to 0.7 m, which is much smaller than the lengths of channel sections and the distance to the observation point.

The following labels are used in the model. l is the distance along the leader channel from differential segment dl to the neutral point. Superscripts $+$ and $-$ correspond to the positive and negative parts of the bidirectional leader. ρ, v, t, and L are the line charge density, leader speed, time, and channel length of the positive or negative part, respectively. The modeling steps are presented below.

- $v^-(t)$ is set to the frame-to-frame speed of the negative end (see Figure 6b of the paper),

- $v^+(t) = 0$,

Table S1: Coordinates of the neutral point, observation point, and the extremities of the positive and negative ends, as seen in Figure S2

Point	Time/Time interval	Coordinates		
		x	y	z
Neutral point	-11.1 to -2.7 ms	0	0	0
Observation point		-0.75	8.4	-4.1
Positive end	-11.1 to -2.7 ms	1.3	0	0
Negative end	-11.1 ms	-1.1	0	-0.4
	-5.5 ms	-2.55	0	-2.1
	-2.7 ms	-2.55	0	-3.1
	0	-2.55	0	-4.1

- $\rho^-(l^-) = k^- l^-$, where k^- is the slope of the negative line charge density distribution along the channel sections, which is varied to match the measured net electric field change,

- $\rho^+(l^+) = k^+ l^+$, where k^+ is the slope of the positive line charge density distribution, which is found from the balance of charge at the positive and negative parts: $\int \rho^-(l^-)dl^- = -\int \rho^+(l^+)dl^+$.

At time t the vertical electric field at ground produced by an L-m long channel whose line charge density is $\rho(l,t)$ can be computed as [S4]:

$$E(t) = \int_0^L \frac{\rho(l,t)H(l)dl}{2\pi\epsilon_0 R^3(l)} = \int_0^L \frac{\rho(l,t)H(l)dl}{2\pi\epsilon_0 (H^2(l) + D^2(l))^{1.5}}, \tag{1}$$

where $H(l)$, $D(l)$, and $R(l)$ are the height of differential channel segment dl and its horizontal and inclined distances to the observation point, respectively.

Applying equation (1) to the positive part and using:

$$\begin{aligned} L^+ &= x^+, \\ H(l^+) &= z_0, \\ D^2(l^+) &= r^2 + (l^+ + x_0)^2, \end{aligned}$$

we obtained the following expression:

$$\begin{aligned} E^+(t) &= \int_0^{L^+} \frac{\rho^+(l^+,t)H(l^+)dl^+}{2\pi\epsilon_0 (H^2(l^+) + D^2(l^+))^{1.5}}, \\ &= \int_0^{x^+} \frac{\rho^+(l^+,t)z_0 dl^+}{2\pi\epsilon_0 (z_0^2 + r^2 + (l^+ + x_0)^2)^{1.5}}. \end{aligned}$$

The negative part is divided into three sections: horizontal, tilted, and vertical. The equation for horizontal section is applied when the length of the negative part is smaller than x_0 ($L^-(t) \leq x_0$) and is derived using equation (1) as follows:

$$\begin{aligned} L^-(t) &= \int_0^t v^- d\tau, \\ H &= z_0, \\ D^2(l^-) &= r^2 + (x_0 - l^-)^2, \\ E^-_{horizontal-section}(t) &= \int_0^{L^-(t)} \frac{\rho^-(l^-, t)H(l^-)dl^-}{2\pi\epsilon_0(H^2(l^-) + D^2(l^-))^{1.5}} \\ &= \int_0^{L^-(t)} \frac{\rho^-(l^-, t)z_0 dl^-}{2\pi\epsilon_0(z_0^2 + r^2 + (x_0 - l^-)^2)^{1.5}} \end{aligned}$$

When $x_0 < L^-(t) < x_0 + 2.1/cos\alpha$, the equation for the tilted section is applied, which is derived as follows:

$$\begin{aligned} L^-(t) &= \int_0^t v^- d\tau, \\ H(l^-) &= z_0 - (l^- - x_0)cos\alpha, \\ D^2(l^-) &= r^2 + ((l^- - x_0)sin\alpha)^2, \\ E^-_{tilted-section}(t) &= \int_{x_0}^{L^-(t)} \frac{\rho^-(l^-, t)H(l^-)dl^-}{2\pi\epsilon_0(H^2(l^-) + D^2(l^-))^{1.5}} \\ &= \int_{x_0}^{L^-(t)} \frac{\rho^-(l^-, t)(z_0 - (l^- - x_0)cos\alpha)dl^-}{2\pi\epsilon_0((z_0 - (l^- - x_0)cos\alpha)^2 + r^2 + ((l^- - x_0)sin\alpha)^2)^{1.5}}. \end{aligned}$$

When $x_0 + 2.1/cos\alpha < L^-(t)$, the equation for vertical section is applied, which is derived as follows:

$$\begin{aligned} L^-(t) &= \int_0^t v^- d\tau, \\ H(l^-) &= z_0 - 2.1 - (l^- - x_0 - 2.1/cos\alpha), \\ D^2(l^-) &= r^2 + (1.8)^2, \\ E^-_{vertical-section}(t) &= \int_{x_0+2.1/cos\alpha}^{L^-(t)} \frac{\rho^-(l^-, t)H(l^-)dl^-}{2\pi\epsilon_0(H^2(l^-) + D^2(l^-))^{1.5}} \\ &= \int_{x_0+2.1/cos\alpha}^{L^-(t)} \frac{\rho^-(l^-, t)(z_0 - 2.1 - (l^- - x_0 - 2.1/cos\alpha))dl^-}{2\pi\epsilon_0((z_0 - 2.1 - (l^- - x_0 - 2.1/cos\alpha))^2 + r^2 + 1.8^2)^{1.5}}. \end{aligned}$$

Equations for the electric fields produced by the positive and negative parts of the bidirectional leader are summarized below.

$$E^+(t) = \int_0^{x^+} \frac{\rho^+(l^+,t)z_0 dl^+}{2\pi\epsilon_0(z_0^2+r^2+(l^++x_0)^2)^{1.5}},$$

$$E^-(t) = \begin{cases} \int_0^{L^-(t)} \frac{\rho^-(l^-,t)z_0 dl^-}{2\pi\epsilon_0(z_0^2+r^2+(x_0-l^-)^2)^{1.5}} \\ \text{if } L^-(t) \le x_0, \\ \int_0^{x_0} \frac{\rho^-(l^-,t)z_0 dl^-}{2\pi\epsilon_0(z_0^2+r^2+(x_0-l^-)^2)^{1.5}} + \\ \int_{x_0}^{L^-(t)} \frac{\rho^-(l^-,t)(z_0-(l^--x_0)cos\alpha)dl^-}{2\pi\epsilon_0((z_0-(l^--x_0)cos\alpha)^2+r^2+((l^--x_0)sin\alpha)^2)^{1.5}} \\ \text{if } x_0 < L^-(t) \le x_0+2.1/cos\alpha, \\ \int_0^{x_0} \frac{\rho^-(l^-,t)z_0 dl^-}{2\pi\epsilon_0(z_0^2+r^2+(x_0-l^-)^2)^{1.5}} + \\ \int_{x_0}^{x_0+2.1/cos\alpha} \frac{\rho^-(l^-,t)(z_0-(l^--x_0)cos\alpha)dl^-}{2\pi\epsilon_0((z_0-(l^--x_0)cos\alpha)^2+r^2+((l^--x_0)sin\alpha)^2)^{1.5}} + \\ \int_{x_0+2.1/cos\alpha}^{L^-(t)} \frac{\rho^-(l^-,t)(z_0-2.1-(l^--x_0-2.1/cos\alpha))dl^-}{2\pi\epsilon_0((z_0-2.1-(l^--x_0-2.1/cos\alpha))^2+r^2+1.8^2)^{1.5}} \\ \text{otherwise.} \end{cases}$$

Frame-to-frame speeds of the negative end were not available before -14.9 ms and were assumed to be equal to the first measurable negative end speed. Extension of the negative leader before -11.1 ms produced only -1.3 V/m change vs. the total field change of 94 V/m. Thus, exclusion of the time interval before -11.1 ms does not materially change any of our results.

In the model, the net electric field change at the ground level is proportional to the charge density slope of the negative end. The proportionality coefficient was estimated to be 2.52×10^8 V×m/C. For the measured net electric field change of 94 V/m between -11.1 and -2.7 ms, the charge density slope was found to be 3.7×10^{-7} C/m^2. The computed and measured electric fields are in reasonably good agreement, as shown in Figure S3, where the contributions from the positive and negative parts are also given.

Figure S4 shows the charge density distribution along the two oppositely charged leader parts at different times, starting from -12 ms with a time step of 4 ms. At -2.7 ms, the line charge density at the negative leader extremity was -1.6 mC/m and the length of the negative leader was 4.4 km. At the

other, positive end of the bidirectional leader, the line charge density was 5.5 mC/m. The contributions of the positive and negative ends to the total electric field at the ground were 310 and -216 V/m, respectively. From -11.1 to -2.7 ms, the charge transfer was 3.3 C, which resulted in an average current of 393 A. If the slope of negative charge density is assumed to remain the same until the leader attachment to ground and the increase of positive end length due to the connection to another floating channel is neglected, the total charge transfer Q will be 5.6 C.

To study the effect of uncertainty in distance measurements, the height of the horizontal channel z_0 (see Figures S2c and S2d) was varied. Variation of the horizontal channel height within ±30% led to less than 13% change in the total charge transfer.

The charge at the positive end is assumed to increase without channel extension, which implies that, for the assumed line charge density profile, the slope of the positive line charge density distribution increases with time. It is likely that the positive-end charge was leaking into the surrounding air via corona discharge on the lateral surface of the channel and via corona streamers at the positive leader extremity. Also, as discussed in the main paper, we cannot rule out a possibility of undetectable steady elongation of the positive end between -8.3 and -2.4 ms.

For comparison, if the positive part of the bidirectional leader were approximated by a time-varying point charge, the charge transfer and average current in the time interval of interest would be 3.0 C and 352 A, respectively. If, additionally, the negative end were assumed to be uniformly charged, the corresponding charge and current values would be 3.6 C and 429 A. All the values predicted by the simpler models are within 10% or so of their counterparts predicted by the more elaborate model presented in Figure S2.

S5. High-speed videos of the bidirectional leader

Two high-speed videos of the bidirectional leader are included in the Supplementary Information. Their legends (descriptions) are given below.

1. Video 1:

   High-speed video from -137 to 0.7 ms showing in slow motion (the 137.7 ms correspond to 20 s) the entirety of the cloud-to-ground lightning discharge, including its initiation, propagation, and ground attachment. The slow-motion frame rate of this video is about 39 frames per second for the first 10 seconds and about 2.5 frames per second for the following 10 seconds.

2. Video 2:

   A magnified view of the positive part of the bidirectional leader between -15.5 and 0.7 ms. The slow-motion frame rate of this video is 2 frames per second.

S6. Supplementary References

[S1] E. R. Williams, Problems in lightning physics – the role of polarity asymmetry, *Plasma Sources Science and Technology* **15** (2), 91 (2006).

[S2] J. Montanya, O. van der Velde, E. R. Williams, The start of lightning: Evidence of bidirectional lightning initiation, *Nature Scientific Reports* **5**, 15180; doi:10.1038/srep15180 (2015)

[S3] O. A. van der Velde, J. Montanya, Asymmetries in bidirectional leader development of lightning flashes, *Journal of Geophysical Research: Atmospheres* **118** (24), 13,504–13,519; doi:10.1002/2013JD020257 (2013)

[S4] V. A. Rakov, M. A. Uman, Lightning: Physics and Effects, Cambridge University Press, New York, ISBN 9780521583275, 2003.
